# Understanding the Influence of Culture on End-of-Life, Palliative, and Hospice Care: A Narrative Review

**DOI:** 10.7759/cureus.87960

**Published:** 2025-07-15

**Authors:** Veena Hira, Sainamitha R Palnati, Saajan Bhakta

**Affiliations:** 1 Department of Epidemiology and Biostatistics, University of Western Ontario, London, CAN; 2 Department of Research, Kansas College of Osteopathic Medicine, Wichita, USA

**Keywords:** culture, demographic, end-of-life care, family medicine, family practice, hospice care, medical practice, palliative care, terminal illness, truth disclosure

## Abstract

Cultural beliefs and values significantly shape end-of-life care decisions, including palliative and hospice care approaches. These cultural influences affect how patients and families view death, pain management, and medical decision-making. This narrative review examines how cultural factors impact care delivery for patients with terminal diagnoses, with particular attention to improving patient-provider communication and trust across diverse populations.

A search of the current literature was conducted from March 1, 2022, to May 1, 2022, through the following databases: PubMed, ScienceDirect, Google Scholar, Directory of Open Access Journals, JSTOR, PsycINFO, ERIC Database (via EBSCOhost), and Academic Search Complete. The following key terms were used with Boolean operations: culture, demographic, end-of-life care, hospice, palliative care, terminal illness, and truth disclosure. A combination of qualitative, quantitative, mixed methods, and review studies was included in the review. The Mixed Methods Appraisal Tool was used to appraise quantitative and qualitative literature critically, and the Risk of Bias in Systematic Review was used to appraise reviews critically.

This narrative review included 25 relevant publications related to influence of culture and patient demographics on end-of-life care, hospice, and palliative care. As each culture has its own unique views on death and dying, it is crucial to note these cultural differences when assisting with end-of-life care to best align with patients’ beliefs and values. Themes such as cultural barriers, communication preferences and family roles emerged from the publications.

The findings of this review highlight the critical role that cultural beliefs and values play in shaping end-of-life care experiences. However, gaps remain in the literature regarding how specific cultural nuances influence care decisions, communication preferences, and family dynamics. Future research should focus on exploring underrepresented ethnic and cultural groups to better understand their unique perspectives on death, dying, and medical decision-making. Additionally, there is a need to develop and evaluate culturally tailored interventions that promote patient-centered care, enhance provider-patient communication, and build trust in end-of-life care settings. Addressing these gaps will help ensure that care is respectful of and responsive to the diverse cultural contexts in which patients and families make these deeply personal decisions.

## Introduction and background

Culture plays a significant role in shaping individuals’ values and decisions throughout life, including how they approach end-of-life care. This narrative review explores how cultural diversity influences patients’ experiences and decision-making in end-of-life, palliative, and hospice care across different regions and diaspora populations. Several important key terms used in this review are defined in Table 1.

**Table 1:** Key terms and definitions

Key terms	Definition
End-of-life care [1]	It is defined as care that entails physical, emotional, social, and spiritual support for patients near the end of life and their families. Such care controls pain and ensures patients are as comfortable as possible. Under this umbrella of end-of-life care are palliative care, supportive care, and hospice care. Palliative care is given to patients with serious or life-threatening diseases to improve their quality of life while reducing pain. It is intended to prevent or treat symptoms of disease and treatment side effects while attending to psychological, social, and spiritual problems. Hospice is a program for patients who are near the end of life. This program offers physical, emotional, and spiritual support for patients and families by ensuring patients are comfortable, symptom-free, and pain-free. Hospice care can be given in various settings (e.g., home, hospice center, hospital, and nursing home)
Patient autonomy [2]	Patients have decision-making capacity and hold the right to make decisions about their health and care, regardless of whether such decisions align with or contradict their health-care providers’ recommendations
Truth disclosure [3]	It consists of relaying truthful information. This is specific to disclosing information that may be uncomfortable or psychologically impact the patient, recipient, and/or healthcare provider(s)
Medical decision-making [4]	It involves a decision derived from a multidisciplinary approach that considers a number of diagnoses and management options, along with the risks, benefits, and alternatives of available options or no treatment. Decision-making in medicine is completed through shared partnership between health-care providers involved in one’s care and the patients themselves or a surrogate decision maker
End-of-life decision making [5]	Medical decisions are made near the end of one’s life. This process necessitates deep consideration of clinical, social, economic, cultural, and religious factors. It is an integral part of palliative and end-of-life care

International perspectives

Cultural values and beliefs around the world deeply influence medical decisions in end-of-life care. For example, in many Asian countries, truth disclosure practices often involve nondisclosure or partial disclosure of terminal diagnoses, reflecting cultural norms centered on protecting patients from distress and showing respect to elders of the patient [6-8]. Family involvement is highly emphasized in decision-making processes, and varying spiritual beliefs shape how death and dying are understood [9-12]. Similarly, Middle Eastern and some Southern European countries integrate faith and spirituality in their views on illness and end-of-life care, as well as incorporating the family in medical decisions [13-17]. Meanwhile, Northern European countries often have opposing views of death and dying, as physicians often prioritize patient autonomy and truth disclosure with the patient over family involvement [18-20]. Across these diverse settings, definitions of illness, death, dying, and palliative care vary widely, influencing how patients and families perceive and make decisions about end-of-life care.

Diaspora perspectives

In multicultural contexts such as North America, diaspora populations navigate a complex interplay between their traditional cultural values and Western healthcare norms. For example, African American communities frequently face systemic racism and healthcare distrust, which impact their engagement with end-of-life care [21,22]. Furthermore, Latin American populations experience additional challenges relating to immigration status, language barriers, fears of deportation, alongside strong family-centered cultural values that shape communication with medical teams [21,23,24]. These unique sociocultural and historical contexts highlight the need for healthcare providers to deliver culturally competent care that acknowledges the ethnic histories and sociopolitical realities of diverse populations. This approach enables medical professionals to foster trust within their patient populations and improve health outcomes.

Common themes across studies

To understand the role of culture in end-of-life care, a comprehensive review of the current literature was necessary. This narrative review offered a snapshot of how culture influences end-of-life decisions worldwide, specifically in East Asia, South Asia, the Middle East, Europe, and North America, while highlighting themes of 1) truth disclosure and communication preferences, 2) patient autonomy and family involvement, and 3) perception of illness and death. A total of 25 studies were included in the review: eight quantitative studies (cross-sectional surveys or quantitative nonrandomized; 32%), nine qualitative studies (interviews or case studies; 36%), and seven reviews (epidemiological, scoping, narrative, or systematic reviews; 28%); one study (4%) was a symposium article.

The studies reviewed emphasize that cultural competence is essential for healthcare providers to understand how traditions, religious beliefs, migrant identities, and social contexts influence patients’ perceptions of illness and what constitutes a “good death.” Family influence on end-of-life decisions is particularly prominent in Middle Eastern, Asian, and Southern European cultures, often within patriarchal family structures [6-9,12,14-16,19]. Attitudes toward palliative care also vary significantly, while it is generally more accepted in North American contexts, some ethnic minorities in Western countries demonstrate stigma toward or misunderstanding of such services [10,24-27]. Central to providing effective end-of-life care is open, culturally tailored communication that respects patients’ and families’ values, facilitated through shared decision-making discussions. Importantly, many studies point to a lack of representation of minority populations, which limits the generalizability of findings and underscores the need for future research to prioritize diverse and marginalized groups [18,21-24,27-29]. Future research is critical to developing culturally sensitive care models that reduce disparities and enhance equity in end-of-life care worldwide. This review aims to understand the importance of integrating cultural considerations into end-of-life care, respecting patient autonomy and family roles, and embracing diverse cultural perspectives to promote more equitable, person-centered care across global and diaspora populations.

## Review

Methods

A comprehensive review of the current literature was undertaken to evaluate, compare, and understand the present-day cultural influences on end-of-life care around the world. Electronic databases were searched from March 1, 2022, to May 1, 2022. Electronic databases included PubMed, ScienceDirect, Google Scholar, Directory of Open Access Journals, JSTOR, PsycINFO, ERIC (via EBSCOhost), and Academic Search Complete.

Search Strategy

The search strategy utilized combinations of keywords and Boolean operators to identify relevant literature. Example search strings include the following: (“culture” AND “end-of-life care”), (“truth disclosure” AND “culture”), and (“hospice” OR “end-of-life care” OR “palliative care” OR “terminal illness”).

Inclusion criteria: Studies were included in the narrative review if they focused on the influence of culture, ethnicity, or demographics on end-of-life, palliative, or hospice care. Included studies explored themes such as truth disclosure, family involvement, patient autonomy, cultural beliefs about death and dying, or health-care decision-making at the end of life. Eligible studies were peer-reviewed and published in English.

Exclusion criteria: Non-English and non-peer-reviewed studies were excluded to ensure data quality and accessibility, though this may have limited the cultural breadth of included studies. Studies identified as conference abstracts, editorials, commentaries, or opinion pieces without primary data were excluded. Studies were excluded from the review if they focused solely on clinical interventions or biomedical outcomes without reference to cultural context.

**Figure 1:**
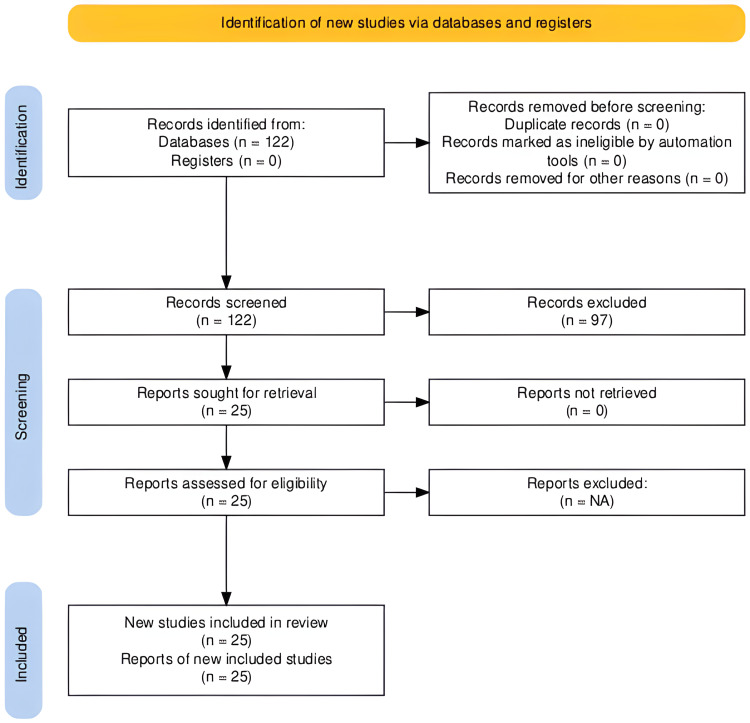
PRISMA flow diagram of the summarized search strategy n: number of studies; PRISMA: Preferred Reporting Items for Systematic Reviews and Meta-Analyses

An initial search yielded 122 publications. After inclusion and exclusion criteria were applied, the final review included 25 studies encompassing a range of study designs, including systematic and narrative reviews, case studies, qualitative observational studies, mixed-methods research, and cohort studies. The utilization of various study designs captured diverse perspectives and experiences on end-of-life care. Figure 1 depicts the search strategy utilized to gain a comprehensive understanding of end-of-life care worldwide.

Data Extraction and Quality Appraisal

Data extraction was performed independently by two reviewers using a standardized form capturing study design, participant demographics, key findings, conclusions, and quality appraisal ratings (Table 2). Quality appraisal was conducted by two researchers, who independently reviewed publications. Since there were various types of literature included in this review, different critical appraisal tools were used. For qualitative and quantitative studies, the Mixed Methods Appraisal Tool was used to critically appraise empirical studies [30]. Based on answers to five questions, a score was provided for each study where one asterisk (*) represented 20% of the quality criteria met, and five asterisks (*****) represented 100% of the quality criteria met [30]. For systematic and scoping reviews, the Risk of Bias in Systematic Review critical appraisal tool was used to critically appraise reviews [31]. Risk of bias was judged based on four domains: 1) concerns regarding specification of study eligibility criteria, 2) methods to identify and select articles, 3) methods used to collect data and appraise studies, and 4) concerns regarding synthesis and findings. This was denoted as "high risk,” "medium risk,” or "low risk." Two studies [13,32] were identified as a symposium article or a systematic review protocol, and thus, risk of bias and quality appraisals could not be conducted. They are denoted as not applicable in Table 2. Discrepancies among the two independent researchers were resolved through consensus meetings.

**Table 2:** Overview of included studies investigating cultural factors affecting end-of-life care communication and decision-making processes ^*^20% of quality criteria met; ^**^40% of quality criteria met; ^***^60% of quality criteria met; ^****^80% of quality criteria met ACP: advance care planning; EOL: end-of-life; MMAT: mixed methods appraisal tool; N/A: not applicable; ROBIS: Risk of Bias in Systematic Review; U.S.: United States of America

References	Country of study	Study design	Population considered	Sample size (n)	Cultural issues highlighted	Key findings and conclusions	ROBIS(MMAT score)	Biases/limitations
Chan et al. [6]	Hong Kong	Qualitative interviews	Health-care providers	131	Denial of death as a cultural taboo impacting end-of-life communication	Sociocultural factors hinder decision-making; authors call for creating government-led policy frameworks	***(60%)	Bias: sampling, social desirability, researcher
Jiang et al. [7]	China	Cross-sectional survey	Cancer patients and families	382 patients, 482 families	Patient vs. family preferences for diagnosis disclosure; timing and setting of disclosure	Patients preferred immediate disclosure, declined for terminal diagnoses; privacy important	****(80%)	Bias: sampling, matching, and response
Li et al. [8]	China	Cross-sectional survey	Oncology nurses	243	Education and work experience influence truth disclosure attitudes	More nurses supported early-stage disclosure than terminal cases; doctors preferred disclosure	****(80%)	Bias: sampling and response
Morita et al. [9]	Japan, Taiwan, Korea	Cross-sectional survey	Palliative care physicians	505 Japan, 207 Taiwan, 211 Korea	National, gender, specialty, and religious differences in disclosure and autonomy attitudes	Japan and Taiwan favored patient-first disclosure; Korea favored family-first; culture shapes autonomy	***(60%)	Bias: sampling and selection; instrument validity limitations
Parsons et al. [10]	U.S., Japan	Cross-sectional survey	Physicians	350 U.S., 362 Japan	Personal attitudes and work culture influence pediatric diagnosis disclosure	U.S. physicians more consistently informed about children; variability in Japan is due to attitudes and culture	***(60%)	Bias: sampling, response, recall, confounding
Tang [12]	China	Cross-sectional survey	Family caregivers	140	Family control over diagnosis disclosure; psychological burden of nondisclosure	Many prefer to withhold diagnosis from elders; uninformed families experience higher psychological stress	****(80%)	Bias: measurement; limited generalizability
Daher [13]	Middle Eastern countries	Symposium article	N/A	N/A	Cancer stigma, myths, and taboos affect information and support needs globally	Despite stigma, people seek information and emotional support; awareness growing	N/A	N/A
Gysels et al. [14]	Europe	Scoping review	N/A	868	Varied cultural views on illness, death, and family role; hospice and euthanasia practices differ	Disclosures differ by culture; family involvement is prioritized in Mediterranean countries	Low risk	Inclusion criteria: Norwegian studies only included English language; other countries’ studies also included studies published in native languages
Montazeri et al. [15]	Iran	Cross-sectional survey	Cancer patients	142	Disclosure impact on patient quality of life	Patients unaware of diagnosis had better physical, social, and emotional functioning	****(80%)	Bias: measurement, sampling
Núñez Olarte and Guillen [16]	Spain	Narrative review	N/A	N/A	Influence of Hispanic traditions on euthanasia, sedation, and diagnosis disclosure	Diverse interpretations of care philosophy; respecting cultural traditions emphasized	High risk	Search strategy not included
Kazdaglis et al. [18]	Global	Narrative review	N/A	234	Balancing full disclosure and withholding information based on patient preferences	Advocates for asking patients about disclosure preferences prediagnosis	Medium risk	Limited depth of analysis
Sprung et al. [19]	Europe	Quantitative nonrandomized	Physicians	3,084	Religious affiliation impacts EOL treatment decisions	Different religions showed distinct treatment limitation patterns and family discussions	***(60%)	Bias: sampling, confounding; limited generalizability
van Bruchem-Visser et al. [20]	The Netherlands	Qualitative interviews	Physicians	14	Societal and cultural mindsets about death and treatment influence overtreatment	“Never giving up” mindset and cultural beliefs drive overtreatment	***(60%)	Bias: sampling; small sample size, limited generalizability
Blackhall et al. [21]	U.S.	Qualitative interviews	Seniors	800	Ethnic differences in disclosure preferences and understanding of truth	Korean- and Mexican-Americans are less supportive of full disclosure compared to African and European Americans; cultural views shape perceptions	***(60%)	Bias: sampling; small sample size, limited generalizability
Davey et al. [22]	U.S.	Quantitative nonrandomized	African American families	12	Culturally adapted intervention improved communication satisfaction	Intervention well-accepted but recruitment slow; anxiety/depression unchanged	*(20%)	Bias: sampling, confounding, small sample size, nonrandomized, inability to stratify in analysis
Kagawa-Singer et al. [25]	U.S.	Epidemiological review	N/A	N/A	Need for culturally competent oncology practices for minority ethnic groups	Emphasizes education, training, and system restructuring for equity	High risk	Search strategy not reported
Searight and Gafford [27]	U.S.	Narrative review	Ethnic minorities	N/A	Differences in communication, decision-making, and advance directives by culture	Family or physician-led decisions are common outside the U.S.; distrust and family roles affect care	High risk	Search strategy not reported
Carrion [23]	U.S.	Qualitative interviews	Physicians	10	Language barriers and cultural beliefs hinder EOL communication with Hispanic patients	Calls for the removal of language barriers and better cultural understanding	***(60%)	Bias: sampling, recall
Smith et al. [24]	U.S.	Qualitative case study	Latino immigrant	1	Barriers include geographic, insurance, fear, language, discrimination	Recommends professional interpreters and conflict resolution	**(40%)	Weak qualitative analysis, small sample size
Raju and Krishna Reddy [11]	India	Qualitative interviews	Glioblastoma patients	31	Death understanding, coping, and preparation vary culturally	Early death preparation advised with caution to avoid harm	****(80%)	Bias: sampling, recall, interviewer
Sinclair et al. [29]	Australia	Qualitative interviews	First-generation Dutch and Italian migrants	30	Migration and cultural identity affect ACP uptake and decision-making	Dutch favored individual decisions; Italians emphasized family roles	****(80%)	Bias: sampling, social desirability
Roman et al. [17]	Romania	Qualitative interviews	Chronically ill Roma patients and caregivers	48	“Silence conspiracy” culture; family controls diagnosis disclosure	Cultural values around dignity and autonomy can cause healthcare conflicts	***(60%)	Bias: sampling, confounding; limited generalizability
Seto Nielsen et al. [26]	Canada	Qualitative interviews	Chinese immigrants	4	Cultural assumptions in palliative care; integration into home routines	Nurses urged to move beyond ethnospecific views to cultural role understanding	****(80%)	Bias: confounding; limited generalizability
Rainsford et al. [28]	Global	Scoping review	N/A	N/A	“Good death” varies culturally but often includes peace and absence of suffering	More data needed for rural perspectives on dying well	Low risk	Fulfilled criteria
Busolo and Woodgate [32]	Global	Systematic review protocol	N/A	N/A	Ethnicity and culture strongly impact EOL communication and decision-making	Recommends cultural competence in health-care provision	N/A	N/A

International perspectives of end-of-life care

Across the globe, there are varying perspectives on end-of-life care that are unique yet share common themes. This section of the narrative review explores each region’s unique preferences in truth disclosure, communication, patient autonomy, family involvement, and perceptions of illness and death. The review begins with a discussion on East Asia, the Middle East, and Europe, then delves into a discussion on diaspora perspectives in regions of the world with increased immigrant communities, such as North America, specifically the United States (U.S.) and Canada.

East Asia

East Asia includes countries such as China, Japan, South Korea, and Taiwan [33] and is characterized by collectivist and family-centered cultural values that strongly influence end-of-life care. Despite differences in language, cultural values, faith beliefs, and governmental policies, these countries share a general emphasis on family involvement in medical decision-making, particularly regarding truth disclosure and patient autonomy. In contrast, South Asia, which comprises countries like India, Pakistan, Bangladesh, and Nepal, has a similarly strong emphasis on family and ethical values but remains underrepresented in end-of-life research and literature [34]. This limited coverage may reflect challenges such as an underdeveloped healthcare infrastructure, future policy frameworks, and cultural barriers to formal palliative care services.

Truth disclosure and communication preferences in East Asia: Communication preferences in end-of-life care are closely tied to cultural norms, the nature of the illness, and the roles of patients, families, and healthcare providers. Studies conducted in China highlight how these preferences, particularly regarding truth disclosure, vary depending on disease stage, patient characteristics, and whether the perspective is that of the patient, family member, or clinician.

Jiang et al. conducted a study in China on the attitudes of patients or families toward truth disclosure and the different stages of cancer. This study found that compared to the families of the cancer patients, cancer patients with a terminal illness had 2.91 times greater odds of wanting to be informed of the diagnosis (95% CI, 2.21-3.85; p < 0.001) and had 1.78 times greater odds than the families of wanting to be informed by the doctor in charge (95% CI, 1.10-2.87; p = 0.02) [7]. When the hypothetical diagnosis changed from early-stage cancer to terminal illness, the number of patients who wanted to know the diagnosis decreased from 90.8% to 60.5%. When families of the cancer patients were asked the same question, the number of families who wanted the patient to know the diagnosis decreased from 69.9% to 34.4%. The attitude toward disclosure was also observed to be influenced by the patient’s sex, as men had 1.52 times greater odds of wanting to know the truth of their illness compared to women (95% CI, 1.01-2.31; p = 0.047) [7].

Disclosure practices are not limited to physicians; nurses in China also navigate complex ethical dilemmas when deciding whether to inform patients of a terminal prognosis. These dilemmas were detailed in Li et al.’s study, which investigated the perspective of Chinese oncology nurses. It was noted that 81% (p < 0.001) of nurses believed patients with early-stage cancer should be informed of the diagnosis, whereas only 44% (p < 0.001) believed patients with terminal illnesses should know the truth [8]. Nurses with higher education levels had 74.2% lower odds (OR, 0.258; 95% CI, 0.079-0.847; p = 0.025) of wanting terminal patients to be informed of their diagnoses compared to nurses with lower education levels. Similarly, nurses with more work experience had 55.2% lower odds of wanting to inform terminal patients compared to nurses with less work experience (OR, 0.448; 95% CI, 0.250-0.804; p = 0.007). On the other hand, nurses with cancer relatives had 4.065 times greater odds (OR, 4.065; 95% CI, 1.350-12.195; p = 0.013) of wanting terminal patients to be gradually told of their diagnoses compared to nurses without cancer relatives were more likely to want terminal patients to be gradually told of their diagnoses. The majority of nurses who preferred to tell terminal patients the truth about their diagnoses also believed that they should be informed by the doctor in charge (73.9%, p > 0.05), immediately after diagnosis (79.5%), and in a quiet room (70.5%, p > 0.05) [8].

These findings highlight the influence of professional experience, personal background, and sociocultural factors on healthcare providers’ perspectives regarding truth disclosure and communication preferences in end-of-life care.

Patient autonomy and family involvement in East Asia: In many Asian countries, physicians tend to adopt a more conservative and family-focused approach to palliative care. However, recent literature has identified a growing emphasis on patient autonomy, with increasing recognition and incorporation of patients’ preferences in clinical practice.

More traditional and conservative attitudes toward death, hospice, and truth disclosure from the caregiver’s perspective were seen in a study conducted by Tang in a hospice care center in China. Results of this survey showed that family caregivers’ death attitudes heavily influenced the process of caring for elders with terminal cancer, which meant cancer patients typically received ineffective communication surrounding their end-of-life issues. Compared to elders who were not aware of their diagnosis, elders who were aware of their diagnosis tended to have a more positive attitude toward death (χ^2^ = 17.9; df = 1; p < 0.001) and were more likely to have their family members around (χ^2^ = 15.9; df = 1; p < 0.001) [12]. On the other hand, elders who were not aware of their diagnosis were more likely to refuse the discussion of death with their family caregivers (χ^2^ = 22.7; df = 1; p < 0.001). Family caregivers of elders who did not know their diagnosis were more likely to have a psychological burden (χ^2^ = 13.1; df =1; p < 0.001).

Morita et al. conducted a cross-cultural survey examining the differences in physicians’ attitudes toward patient autonomy and a good death in East Asian countries (i.e., Japan, Taiwan, and Korea), and found that, compared with historical data, patient autonomy was being considered more often in these countries. In this study, intercountry differences in physicians’ attitudes toward patient autonomy were found. For example, Japanese physicians had 1.7 times greater odds (OR, 1.7; 95% CI, 1.0-2.7; p = 0.006), and Taiwanese physicians had 4.9 times greater odds (OR, 4.9; 95% CI, 2.4-9.8; p < 0.001) of telling the patient first of malignancy, then the family, compared to Korean physicians [9]. Moreover, Japanese physicians had two times greater odds (OR, 2; 95% CI, 1.4-3.1; p < 0.001) of wanting to inform the patient of malignancy, even if the family disagreed, compared to Korean physicians.

Together, these findings illustrate a gradual but meaningful shift toward greater respect for patient autonomy in Asian palliative care. They underscore the ongoing need for culturally sensitive clinical practices that balance patient-centered communication and family-centered values in these settings.

Perception of illness and death in East Asia: Perceptions of illness and death in Asian societies are deeply shaped by cultural traditions, such as Confucian filial piety in Korea and norms of nondisclosure and deference to authority in Japan [9]. However, recent shifts, particularly in Taiwan, indicate a growing emphasis on patient autonomy in end-of-life care. The previous study by Morita et al. found that physicians’ cultural backgrounds significantly influenced their views of a “good death” for the patient. Taiwanese physicians, for example, placed greater importance on life completion compared to their Japanese and Korean counterparts (effect sizes = 0.22 and 0.33, respectively), and valued being free from tubes and machines more highly (effect sizes = 0.15 and 0.23) [9]. Conversely, humor was regarded as significantly less important by Taiwanese physicians (effect size = 0.21 for both Japan and Korea). Korean physicians were more likely to prioritize being cognitively intact at the end of life (effect size = 0.30), whereas Japanese physicians placed greater value on contributing to others (effect size = 0.25). The relatively lower emphasis on preparation for death among Japanese physicians may be rooted in traditional beliefs, such as the notion that "pokkuri" (sudden death or unawareness of impending death) constitutes a good death [9].

Although palliative care and end-of-life treatment are generally respected in East Asian countries, structural and cultural barriers still persist. In Hong Kong, for instance, Chan et al. identified several gaps in the development of palliative care, noting that the quality of dying remained below that of other high-income nations. Interviews with healthcare providers revealed that sociocultural factors, such as the cultural taboo surrounding death, contributed to communication barriers in end-of-life discussions [6]. Physicians may hesitate to discuss prognosis or care options due to fears of violating cultural norms. Filial piety further complicates care, as families often feel obligated to pursue aggressive treatments to prolong life, even when such interventions may not align with the patient’s wishes [6]. Despite this, some patients expressed a preference for comfort care over life-prolonging interventions.

From the patient's perspective, views on death and dying are also shaped by the illness trajectory and psychosocial context. Raju and Krishna Reddy explored the experiences of glioblastoma (GBM) patients in Bengaluru and found that their rapid decline and unmet psychosocial needs intensified fears surrounding death. Death itself was not considered a taboo topic in these conversations; rather, it was the topic of God [11]. Patients’ perceptions of dying were deeply influenced by cultural and spiritual beliefs, prior experiences, and individual personality traits. Many patients coped through spiritual reflection, while others derived comfort from being treated with respect and having their everyday needs met. This study reinforced previous research emphasizing the need for early preparation for death and dying among GBM patients during hospitalization [11].

Overall, perceptions of illness and death across Asian countries are shaped by an interplay of cultural, religious, and familial values. While traditional norms, such as filial piety in Korea, death denial in Hong Kong, and nondisclosure practices in Japan, continue to influence communication and care preferences, there is growing evidence of a cultural shift toward greater consideration of patient autonomy, particularly in Taiwan. These evolving attitudes highlight the importance of developing palliative and end-of-life care models that are both culturally sensitive and responsive to individual patient and family values. As healthcare systems in the region continue to adapt, balancing longstanding social expectations with patient-centered care remains a key challenge and a critical opportunity for improving the quality of end-of-life experiences.

Middle East

The Middle East comprises countries around the southern and eastern shores of the Mediterranean Sea and the Arabian Peninsula [35]. In Middle Eastern countries, traditional family structures and strong religious and cultural beliefs significantly influence end-of-life care decisions. These cultural factors shape both the patient’s and physician’s approach to issues such as truth disclosure and care responsibility.

Truth disclosure and communication preferences in the Middle East: The study by Montazeri et al. in Iran explored the impact of cancer diagnosis disclosure on quality of life. The findings revealed that patients who knew their diagnosis had lower physical (M = 74.7, SD = 19.4, p = 0.001), emotional (M = 60.2, SD = 23, p = 0.014), and social functioning (M = 75.5, SD = 24.2, p < 0.001), but higher levels of fatigue (M = 34.5, SD = 24.8, p = 0.014) and financial difficulties (M = 58.3, SD = 38.4, p = 0.005) compared to those who were not informed [15]. Additionally, a statistically significant relationship was seen between illiteracy and patients who were unaware of their diagnosis (74.3%, n = 55, p = 0.001). These findings align with other studies from countries with traditional cultural backgrounds, where physicians often avoid disclosing diagnoses to protect the patient from psychological distress. The study concludes that cancer disclosure guidelines should vary according to cultural norms and available healthcare resources. In countries with traditional cultural backgrounds, like those in the Middle East, physicians should consider cultural preferences when deciding whether to disclose a diagnosis, as this can significantly impact the patient’s experience and quality of life [15].

Patient autonomy and family involvement in the Middle East: In Middle Eastern cultures, the family plays a pivotal role in caregiving, with institutional care such as nursing homes or hospices being rare. This cultural expectation places considerable responsibility on family members, particularly women, who frequently serve as the primary caregivers throughout the patient’s illness and end-of-life period [13,15].

Perception of illness and death in the Middle East: The study by Daher focused on the cultural beliefs and values in cancer patients, specifically in Middle Eastern countries. The study highlighted that patients who relied on spirituality and faith during their illness experienced more positive outcomes, such as reduced depression, longer survival, and fewer postsurgical complications. Conversely, those with negative spiritual thoughts exhibited heightened pain sensitivity. The study also addresses various cultural myths about end-of-life care, such as the belief that disclosing a diagnosis to the patient may hasten death. Daher calls for health professionals to understand and respect the diverse social and cultural aspects of life and death in the Middle East, emphasizing the need for culturally sensitive care [13].

Europe

Among the European population, there is a rich diversity of ethnicities, cultures, religions, and languages, reflecting the continent's complex sociocultural landscape [36]. Europe is home to approximately 160 distinct cultures, each of which can influence values, beliefs, and practices surrounding illness, death, and dying [36]. This cultural heterogeneity gives rise to considerable variation in end-of-life decision-making across European countries. For example, preferences for truth disclosure, the use of life-sustaining treatments, and attitudes toward palliative sedation or assisted dying may differ widely based on national policies, religious beliefs, and family structures. In some countries, patient autonomy and advance care planning (ACP) are strongly emphasized, while in others, decision-making may be more collective and involve close family members. Understanding these variations is essential for delivering culturally competent end-of-life care that respects individual and community values across the diverse populations of Europe.

Truth disclosure and communication preferences in Europe: Disclosure practices at the end of life vary significantly across Europe, with notable differences observed between Northern and Southern countries. Physicians in Southern European countries (i.e., Italy, Spain, and Portugal) tended to practice partial disclosure, often withholding prognostic terminal information from patients, a practice that conflicted with the ethical obligation to maintain open and honest communication about a patient’s diagnosis [14]. Similarly, the Roma community was opposed to informing terminal patients about their condition [17]. The Roma cultural background and views, as well as language barriers and a lack of physicians’ time, were the main issues in providing adequate end-of-life care. These may lead to further ethical dilemmas and moral distress if not understood and addressed. Roman et al. further recognized poor communication among the medical team with Roma patients and the next of kin. For the Roma community, understanding of dignity is closely related to their cultural practices, and by ignoring these cultural practices, conflicts between providers and the Roma community could be created [17]. These findings highlight the critical need for culturally sensitive communication strategies in end-of-life care across Southern Europe, especially when working with communities like the Roma, whose traditional values and disclosure preferences may differ from those of other European cultures.

Traditionally, attitudes toward truth disclosure in other Southern European countries, such as Spain, have differed significantly from those in Northern Europe (i.e., Norway, Belgium, Germany, and the Netherlands) and Anglo-Saxon contexts. Núñez Olarte and Guillén noted that Spanish culture has historically favored more protective approaches, often limiting the amount of diagnosis information shared directly with patients. These practices contrast with the Northern European attitude toward truth disclosure, which emphasizes patient autonomy and shared decision-making [14,16]. Furthermore, Gysels et al. observed that Southern European countries did not initially align with the Western norm of open communication between physicians and patients. However, more recent trends indicate a gradual shift: healthy populations in Southern European countries are increasingly integrating more transparent dialogue and informed disclosure from healthcare providers, suggesting a cultural transition toward more open clinical communication [14].

Additionally, in a cross-national study of European intensive care units (ICUs), Sprung et al. found substantial religious differences in end-of-life care practices, which were influenced by the cultural and religious practices of the physicians. Information about patients’ end-of-life wishes was only available for 26% of cases and was obtained for physicians who identified as the following: Protestant (30%), Greek Orthodox (6%), Jewish (11%), or unaffiliated with a religion (27%) [19]. In contrast, Catholic (25%) and Muslim (0.25%) physicians reported lower recognition of patients’ end-of-life. The authors attributed this difference to the greater respect for patient autonomy in the North, where more Protestant practicing physicians are found, compared to the relatively low numbers of Catholic physicians in the South, who tend to operate on more paternalistic attitudes [19]. These findings highlight significant variability in truth disclosure and communication preferences, which appear to be influenced by the religious and cultural values of physicians and patients.

Patient autonomy and family involvement in Europe: Family involvement plays a notably different role in palliative and end-of-life care across Europe, reflecting broader cultural divides between Southern and Northern European countries. In Southern European countries, collective decision-making and familial responsibility are often prioritized over patient autonomy, particularly in regions with strong Latin cultural influences and Mediterranean areas [14,16]. In many cases, families in these settings assume full responsibility for decision-making, which can result in the minimization of the patient’s own voice in their care [14,16,17]. The presence and influence of family members also affect disclosure practices, as seen in Roma communities, where physicians often follow the wishes of the next of kin rather than those of the patient [17]. Within this community, paternalism is also considered a benefit to the Roma community, as strong paternalistic actions were seen as appropriate and justified in healthcare decisions to prevent harm to the patient [17].

By contrast, Northern European countries such as Germany and Belgium tend to emphasize individual autonomy, with patients more likely to exclude family members and nonmedical professionals from end-of-life decisions [14]. However, there are exceptions to this general trend. For example, Norwegian physicians typically retain ultimate responsibility for treatment decisions but often engage the patient’s family in the decision-making process, which was shown to promote greater consensus around treatment options [14].

Furthermore, the study conducted by Sprung et al. found that discussions of end-of-life decisions with patients’ families varied according to the physician’s religion and regional norms. Differences were seen in whether family members were consulted, the timing of those discussions, and the reasons given for not including families [19]. For example, discussions with the family took place more often if the physician was Protestant (80%), was Catholic (70%), had no religious affiliation (66%), or was Jewish (63%) compared to Greek Orthodox (33%) or Muslim (25%). Greek Orthodox (76%), Muslim (56%), or Catholic (47%) physicians were more likely to cite “lack of patient responsiveness to therapy” as a reason for lack of discussion with the family, compared to Jewish (42%), Protestant (31%), or physicians with no religious affiliations (30%) [19]. This suggests that the role of the family in end-of-life decision-making is not only culturally mediated but also influenced by the personal beliefs of the healthcare provider. Overall, the role of the family in end-of-life care varies considerably across different European cultures, shaping how decisions are made and whose voices are prioritized in the process.

Perception of illness and death in Europe: Across Europe, perceptions of illness, death, and dying vary widely due to diverse spiritual, religious, and cultural values. For example, end-of-life decisions in European ICUs reflect these variations, particularly in relation to physicians’ religious affiliations, as detailed in the study by Sprung et al. It was found that Catholic (59%), Protestant (49%), and Muslim (37%) physicians reported higher rates of treatment withdrawal compared to Jewish (19%) and Greek Orthodox (22%) physicians [19]. Furthermore, multivariate analysis revealed that the odds of withdrawing therapy (including active shortening of the dying process) were also shaped by religious affiliation and regional culture. The odds of withdrawing therapy were 1.34 times higher for Catholic physicians (OR = 1.34, 95% CI = 1.09-1.64, p = 0.005), 79% lower for Jewish (OR = 0.21, 95% CI = 0.15-0.29, p < 0.0001), and 77% lower for Greek Orthodox physicians (OR = 0.23, 95% CI = 0.15-0.35, p < 0.0001). These decisions were shaped by both physicians' and patients' religious backgrounds, as well as broader regional cultural differences. For example, Jewish physicians withdrew treatment more frequently in Northern (36%) than in Southern (6%) Europe [19]. In Southern Europe, cardiopulmonary resuscitation (CPR) was more frequently performed (53%) and treatment limitations were less common (47%) when the physician and patient had different religious affiliations, compared to cases where they shared the same affiliation (29% CPR, 71% limitations; p < 0.001) [19].

This variation in end-of-life practices is further illustrated by specific national cultural values, such as those found in Spain. Núñez Olarte and Guillen noted how Spanish culture influenced attitudes toward pain relief and sedation as opposed to euthanasia. In accordance with Spanish culture, interventions intended to accelerate death or kill the patient are considered morally wrong; however, using sedation for physical or spiritual pain relief is supported [16]. Using sedation to achieve unconsciousness was often perceived as the best way to die and may also be the reason palliative care has been so well received in Spain. Additionally, the concept of agonia is present in Spanish culture, defined as the state prior to death in instances where life extinguishes gradually. An in-depth understanding of this cultural concept can serve as a useful tool for palliative care teams in end-of-life care, enabling time for families to prepare to witness the death of their relative [16]. Information and diagnosis disclosure is another aspect of the Spanish palliative care movement, as certain diagnoses are not always relayed to the patient. This is due to the fact that respect for individual and cultural differences in attitudes toward death and dying is considered an essential topic of deliberation when deciding appropriate care for terminally ill patients [16].

While Southern European cultures like Spain emphasize spiritual comfort and family-centered care, Northern European countries reflect more clinical approaches, shaped by legal, religious, and societal frameworks. For instance, the study conducted by Van Bruchem-Visser et al. focused on the more clinical views that Northern Europeans tended to have regarding end-of-life treatment. The authors conducted a qualitative study on Dutch physicians in which mechanisms that influenced requests for futile suffering and death were found to be one of the main drivers of overtreatment [20]. The patients’ specific views on the meaning of suffering and death were often difficult for the physician to accept. In Dutch culture, the medical perspective often dominates the decision-making process, and as such, physicians may focus on the medical aspects of disease and neglect the social and cultural well-being of the patient. In this study specifically, the medical view was used by physicians as a tactic for refusing interventions of multidisciplinary consultation of social, mental, spiritual, cultural, and ideological aspects for patients’ well-being [20]. The physician often found the interventions patients requested were, in fact, medically futile. The authors argue that this tendency reflects a broader cultural pattern in which physicians may overlook or undervalue the role that patients’ cultural beliefs play in shaping end-of-life preferences [20].

This emphasis on clinical judgment over culturally nuanced care aligns with broader attitudes seen in other Northern European countries, such as the Netherlands. This includes the practice of euthanasia, which is more widely accepted in The Netherlands and Belgium due to longstanding norms of medical autonomy [14]. These societal values have significantly influenced both national policy and clinical practice. In particular, euthanasia and physician-assisted suicide have been accepted in Dutch society for over a century. Ethnographic research further suggests that euthanasia requests often carry symbolic meaning, creating opportunities for open dialogue about death, suffering, and the patient [14].

Meanwhile, in Belgium, greater availability of palliative care has been linked to a reduction in euthanasia requests, although not completely avoidable. The review by Gysels et al. cited a study that found 90% of physicians in Belgium reported significantly fewer cases of euthanasia and assisted suicide (1,917 vs. 10,319), with most occurring among patients with nervous system disorders and predominantly in hospital settings [14]. In contrast, active euthanasia remains illegal in Germany, where the National Board of Physicians opposes any liberalization of euthanasia laws. Despite Germany’s secular and individualistic social values, public acceptance of euthanasia remains relatively low. Similarly, in Norway, both euthanasia and physician-assisted suicide are illegal and explicitly condemned in the Norwegian Medical Association’s ethical guidelines. Norway’s Lutheran heritage and influence from a Puritan “moral minority” have shaped national discourse, leading to more conservative physician attitudes toward treatment limitation and euthanasia compared to other Western countries. A strong cultural respect for the law has also contributed to the low incidence of hastening death, even among physicians with more liberal views. Only recently has Norway begun to distinguish between palliative sedation and euthanasia, with formal guidelines now in place [14]. Spain, although a country that still holds more conservative views over end-of-life care, has seen a recent increase in acceptance of euthanasia among the general population. Other Southern European countries, such as Italy and Portugal, still remain some of the countries with the lowest acceptance of euthanasia [14].

In parallel with the dividing views of end-of-life care between Northern and Southern European countries, Sinclair et al. demonstrated that first-generation Dutch and Italian immigrants in Australia shared similar opposing views of such care. Diverse views were expressed, which were informed by their cultural heritage and experiences as migrants. Dutch participants likened ACP discussions to euthanasia and also had a more individualistic approach to the conversations surrounding medical decision-making, specifically regarding personal autonomy in reference to decision-making, as autonomy and self-determination are recognized characteristics associated with Dutch culture [29]. On the other hand, Italian culture speaks mainly to familial roles and emphasizes a family-based decision-making style. This gave Italian migrants the perception that advanced care planning conversations were unnecessary and that they preferred traditional, home-based approaches to caring for older family members [29].

These studies have shown that perceptions of illness, death, and dying reveal clear contrasts between Northern and Southern European countries. Southern European cultures often prioritize spiritual comfort, family-centered care, and a more conservative approach to end-of-life interventions, while Northern European societies emphasize the medical autonomy of the patient in decision-making. Despite these traditional distinctions, attitudes are gradually evolving in these countries. Increasing openness to patient-centered communication and shifts in ethical frameworks suggest a growing convergence of perspectives. Understanding the emerging view of end-of-life care is essential for delivering sensitive, individualized end-of-life care.

Diaspora perspectives

The continent of North America covers over 9.35 million square miles with significant ethnohistory and a variety of cultures in both rural and urban settings [37]. This review primarily focuses on the U.S. and Canada, as there exists a gap in research studying other countries in the North American continent, such as the West Indies, Central America, and Mexico [37]. A high proportion of the population of North America belongs to various diasporas, communities formed by individuals who have migrated from their ancestral homelands, yet continue to maintain strong cultural, social, or emotional connections to their origins [38]. These communities, along with other ethnic minority populations, offer unique perspectives on end-of-life care that are shaped by their cultural values, religious beliefs, and lived experiences. As such, it is critical to consider the needs of these populations in the delivery of palliative and end-of-life services, particularly in multicultural regions like the U.S. and Canada.

Truth Disclosure and Communication Preferences in North America

In multicultural contexts such as the U.S. and Canada, truth disclosure at the end of life should consider the diverse cultural beliefs and communication norms of the patient. With roughly one-third of the U.S. population identifying as ethnic, Searight and Gafford called for physicians to understand how cultural backgrounds influence patients’ and families’ responses to serious illness and approach end-of-life decisions. While Western biomedical ethics emphasize openness and patient autonomy, in many non-Western cultures, directly discussing terminal illness is considered taboo or potentially harmful, as it can evoke anxiety or depression in the patient [27].

A cross-cultural study by Parsons et al. compared attitudes toward truth disclosure and communication among oncologists in the U.S. and Japan; 98% of U.S. physicians surveyed agreed that it was their responsibility to inform the patient of a cancer diagnosis [10]. Most U.S. physicians also agreed that the child’s knowledge of their diagnosis would encourage participation in their care (99.1%) and improve psychosocial support through open communication (98.3%). In contrast, Japanese physicians were more likely to defer disclosure of the illness to the patient’s family (16.3% Japan vs. 6.9% U.S., p < 0.0001) and fear that community knowledge of the illness would lead to social stigma and isolation (63.3% Japan vs. 6.6% U.S., p < 0.0001) [10]. These contrasting views reflect broader cultural values: U.S. physicians were more driven by a professional sense of responsibility for disclosure, whereas Japanese physicians were more influenced by family roles, social harmony, and cultural norms [10]. Among Asian diaspora communities in North America, such values may continue to shape disclosure preferences even within Western healthcare systems.

The review by Searight and Gafford further highlighted important communication challenges for family physicians providing end-of-life care to ethnically diverse patients [27]. The authors noted that in some cultures, physicians often used softened language to shield patients from the emotional burden of a terminal diagnosis. For example, when communicating bad news, many Japanese and African physicians preferred using terminology that lessened the impact of severity, such as “growth,” “mass,” or “unclean tissue,” rather than directly describing a terminal condition [27]. Similarly, in Hispanic, Chinese, and Pakistani communities, families often took an active role in protecting patients from distressing information. In multicultural North American settings, this may manifest as family members selectively translating or withholding diagnostic or treatment details from the patient. These patterns underscore how truth disclosure continues to be shaped by cultural norms and family dynamics, even in settings far removed from individuals’ countries of origin.

While cultural norms continue to influence truth disclosure practices across generations, lived Native Americans, for example, hold specific views on truth disclosure and the spoken word. A common Navajo belief is that negative words and thoughts about health become self-fulfilling if spoken aloud [27]. Navajo people place significant value on thinking and speaking in a positive way, and thus, Navajo informants are reluctant to discuss advance directives or anticipated therapeutic supports with patients as these verbal exchanges were potentially portrayed as injurious to the patient’s health [27]. Similarly, some Asian cultures are also reluctant to discuss possible death as there is a common belief that direct acknowledgment of mortality may be self-fulfilling. Another population, African Americans, also holds a unique perspective on healthcare and dying, rooted in a history of systemic injustice and medical mistrust in Western medical institutions [21]. In a comparative study of Korean, Mexican, African, and European American participants, Blackhall et al. found that African and European Americans were more likely to support full disclosure of a terminal diagnosis, grounded in the view of selfhood as being autonomous. However, disclosure was also described as emotionally difficult, creating tension between respect for self-agency and avoidance of emotional harm. In contrast, Korean and Mexican American participants were significantly less likely to support direct disclosure and often described truth disclosure as a more nuanced process, preferring to use vague language or contextual clues, rather than explicit communication [21]. Blackhall et al. attributed these differences to varying conceptions of selfhood: African and European Americans tended to view the self as autonomous and were proud to make individual choices, which could be portrayed as dehumanizing if the ability to make choices about oneself was taken away. Meanwhile, Korean and Mexican Americans emphasized relational identity and expected family members to act on the patient’s behalf, as they often viewed themselves as part of a larger group [21]. The study further underscored the importance of cultural sensitivity while cautioning against stereotyping, emphasizing that individual preferences cannot be assumed based on group identity alone.

Further cultural nuances emerged in a qualitative study conducted in South Florida by Carrion, which examined how physicians navigated terminal diagnoses and hospice referrals when working with Hispanic patients. Physicians expressed uncertainty in effectively communicating with Hispanic families, particularly when they perceived a cultural expectation to involve the extended family in sensitive discussions [23]. Language barriers and differences in how illnesses are interpreted added further complexity to the disclosure process. For instance, physicians noted that some Hispanic patients, particularly those from rural areas, held beliefs that Western medicine could cure all diseases, which contributed to hesitancy around hospice and at-home care.

Similarly, Smith et al. conducted a case study on a young Latino immigrant woman with terminal cancer to explore the cultural considerations involved in end-of-life care. The case underscored the importance of familismo, machismo, and fatalismo, which are values that influence how truth is communicated and by whom [24]. Machismo, in this case, leads male relatives to dominate decision-making, limiting direct communication with the patient herself. These culturally specific communication preferences can delay care or reduce engagement with hospice services. The authors argued that recognizing such values is essential to effective, compassionate care and that open, culturally responsive dialogue is needed to build trust [24]. Taken together, these findings demonstrate that truth disclosure at the end of life is not a one-size-fits-all situation. Rather, it is deeply embedded in broader cultural, familial, and historical contexts. Open, respectful communication that is attuned to both the patient’s cultural background and individual preferences is essential to providing compassionate and effective care.

Patient Autonomy and Family Involvement in North America

In diaspora settings, patient autonomy and family involvement often exist in tension, influenced by both cultural expectations and medical norms of the host country. In most Western healthcare systems, informed consent and independent patient decision-making are encouraged, except in circumstances where the patient is deemed psychologically unfit or seriously impaired by a medical illness. However, Carrion noted that Hispanic patients often prioritized collective decision-making, with family members playing an active and protective role. Physicians in this study expressed uncertainty about how to navigate these expectations while still honoring institutional protocols that emphasize patient autonomy [23]. This uncertainty was further compounded by a lack of cultural competency training, which left some providers unprepared to effectively involve families in a culturally respectful manner [23].

Similarly, the case study presented by Smith et al., involving a young Latina immigrant with terminal cancer, highlighted the influence of family-centered values, particularly familismo, on decision-making. The patient’s extended family played a central role in navigating medical care, reflecting a collectivity orientation that may contrast with individualistic models common in Western systems [24]. The aforementioned gender roles also shaped family involvement: machismo often placed medical authority and final decisions in the hands of male relatives, even when the patient was cognitively capable. These findings challenge assumptions about autonomy as a universal principle in Western healthcare and underscore the need to accommodate diverse models of agency in end-of-life care.

Furthermore, Searight and Gafford noted that Korean and Mexican Americans were more likely to involve family members in medical decisions compared to European or African Americans. Additionally, some Asian cultures typically view physicians with a high degree of authority and respect, and would typically defer medical decisions to expert judgment [27].

In a complementary vein, Kagawa-Singer et al. provided a comprehensive definition of culture at each stage in a cancer patient’s journey and noted that African Americans also tended to include their family in medical decisions, whereas their White counterparts were more likely to exclude family members from end-of-life discussions [25]. Recognizing that both cultural and systemic factors affect the quality of cancer care, the authors advocate for open discussions about patients’ wishes at each stage in a cancer patient’s journey should be openly addressed between caregiver and patient.

Another critical issue brought to attention by Searight and Gafford was advanced directives, which are legal documents specifying preferred medical treatment if the patients are no longer capable of decision-making. Advance directives were found to be significantly less likely to be completed in Asian, Hispanic, and African American patients [27]. Among African Americans, reluctance toward advanced directives was linked to a historical legacy of segregation and a perception that do-not-resuscitate orders limit access to healthcare. Additionally, African American patients and physicians viewed suffering differently from their White counterparts, seeing suffering as spiritually meaningful and adding value to life, as well as their perseverance through suffering and pain as a demonstration of religious faith. In Hispanic communities, the lack of advance directives is thought to stem from the view of collective family responsibility. For this population, a collective-oriented decision-making approach was found to be the optimal solution for the patient and family [27].

Kagawa-Singer et al. highlighted that culture is a prime factor in the persistence of health disparities in the U.S. and should be addressed to promote equity in access and quality care for cancer patients [25]. The authors posit that the reason cancer incidence rates vary significantly across cultural groups within the U.S. is that each cultural group has special advantages or disadvantages. In terms of end-of-life care, it is seen that African Americans have less utilization of advanced directives and stronger beliefs in vitalism compared with non-Hispanic Whites. Vitalism is rooted in African American spirituality as well as enforced in this diaspora through centuries of abuse and discrimination within medical care. This has led to mistrust in the dominant medical establishment and fear of having their lives taken away from them prematurely. This also means that African Americans are more likely to push for intensive treatment than to consider themselves terminally ill. African Americans were also seen to request more spiritually focused information compared to non-Hispanic White participants, as they valued the protection of life at all costs and requested spiritual guidance to support the extended family in medical decisions [25].

Recognizing these dynamics, Davey et al. argued that interventions designed for White populations may not adequately address the cultural needs of African American patients and families in end-of-life care. In a pilot intervention, the authors evaluated the effectiveness of a culturally adapted program to improve communication between African American parents with cancer and their children [22]. Parents who completed the culturally adapted intervention showed significant improvement in their communication with their children (p = 0.056, r = 0.60), compared to those who completed the normal psychoeducation intervention [22]. Children who completed culturally adapted family interventions were more satisfied (p = 0.006, r = 0.62) compared to children who completed the normal intervention. This intervention drew upon Afrocentric coping strategies, such as reliance on faith, community support, and role flexibility, and created space for open dialogue. The authors suggested that cultural alignment, along with delivery by racially matched facilitators, enhanced participant engagement and emotional safety. Importantly, the program reflected a core value in African American communities: collective coping and prioritization of group over individual needs [22].

Together, these studies demonstrate that autonomy and family involvement should not be treated as mutually exclusive ideals, and instead should be navigated in ways that reflect patients’ and families’ cultural backgrounds and social realities. A culturally responsive approach to end-of-life requires flexibility in how the agency is understood and enacted.

Perception of Illness and Death in North America

Among immigrant and minority communities in North America, definitions of illness and dying are deeply rooted in cultural and spiritual traditions, while also shaped by interactions with healthcare institutions and historical patterns of care. For instance, Carrion found that some Hispanic patients viewed terminal illness through a religious or fatalistic lens, believing that suffering and death are predetermined or part of a divine plan. These beliefs sometimes led to a delayed acceptance of palliative care or hesitancy toward hospice referrals [23]. Likewise, the theme of fatalismo was described in Smith et al.’s case study as a pessimistic attitude toward the future and manifested as delayed treatment-seeking and resignation in the face of a terminal diagnosis [24]. Such beliefs reflect a different orientation to suffering and healing compared to Western views, which may not align with the clinical team’s focus on prognosis and intervention. Understanding these important cultural values, as well as appropriately addressing patients' fears regarding documentation status, would allow physicians to establish trust with the patients and offer optimal care [24].

Seto Nielsen et al. further explored Chinese immigrant communities in Toronto, Canada, particularly in how advanced cancer patients of this demographic conceptualized palliative home care. Using a postcolonial lens, the authors found that the perception of palliative home care as being an invasive or stigmatizing form of support was common, not necessarily due to cultural differences but due to systemic assumptions held by healthcare providers [26]. Specifically, the authors questioned whether the healthcare providers placed too much emphasis on culture when caring for immigrants, as the cultural assumptions made by healthcare providers often presented as stereotypes and were thus identified as barriers to patient-centered care at the end of life for immigrant patients. Overall, the findings from this study showed the need for healthcare providers to acknowledge their own cultural assumptions to adopt an appropriate care plan for the patient [26].

Finally, Rainsford et al. broadened the conversation on end-of-life care by conducting a systematic review exploring how rural populations around the world, which are often culturally distinct and underrepresented, define a “good death.” The majority of rural residents preferred to die at home or in their rural community, some even going as far as forgoing medical interventions in favor of dying in their homes or communities [28]. Religiosity was also more prevalent in rural populations, thus shaping their views on a “good death.” For example, in populations where Christianity was prevalent, concepts of "good" and "bad" were aligned with views surrounding death and the afterlife, as they had a significant impact on the health and well-being of the community. For rural populations in developed countries, a "good death" was viewed as placing greater emphasis on patient autonomy and minimizing a sense of struggle, whereas rural residents in nondeveloped countries viewed good deaths as ones that do not disrupt the life and health of the community [28]. These findings suggest that perceptions of death were not only culturally constructed but also varied geographically.

Collectively, these findings show the need for end-of-life care in Western settings to be responsive to diverse worldviews and grounded in patients’ social realities. Attending to these nuances can foster trust and improve alignment between palliative medical care and what patients and families consider meaningful at the end of life.

Discussion

Comparative Analysis of Studies From Around the World

The studies considered in this review illustrate how cultural frameworks inform end-of-life care practices across both international and North American multicultural contexts. Cross-regional comparisons demonstrate significant variation in approaches to truth disclosure, autonomy, and the meaning attributed to death and dying. Importantly, many of these cultural values are retained among immigrant and minority populations in Western healthcare systems, influencing clinical interactions and decision-making. Synthesizing insights from both global and diaspora-focused research enables a more nuanced understanding of how end-of-life care is shaped by an intersection of cultural, social, and systemic factors. This discussion interprets the findings from the studies included in this review in relation to the three core themes: 1) truth disclosure and communication practices, 2) models of autonomy and family involvement, and 3) cultural and faith-based understandings of illness and death.

Truth disclosure: Truth disclosure at the end of life is a complex ethical practice shaped by cultural, institutional, and relational contexts. While dominant Western bioethics frameworks tend to prioritize autonomy and transparency, emphasizing patients’ rights to know about their illness, these assumptions do not hold universally. In many non-Western societies, direct disclosure may be considered harmful, particularly if it risks causing emotional distress, disrupting family harmony, or undermining the spiritual understanding of death.

Systematic and narrative reviews [18,28,32] illustrated that truth disclosure practices vary not only between but also within nations, influenced by sociocultural norms, religious beliefs, and healthcare systems. Importantly, these studies argue that ethical communication must be evaluated from both patient and provider perspectives. For instance, Kazdaglis et al. showed that patients may interpret clinical bluntness or strictly factual communication as cold or dehumanizing, especially when bad news is delivered without accompanying emotion, relation, or spiritual support. In such contexts, even accurate disclosure can cause distress if it is misaligned with the patient’s expectations about how difficult truths should be conveyed [18].

These findings call into question the embedded Western norms that full, immediate transparency of a diagnosis is preferred and expected by the patient [39]. Rather, they call for a more nuanced approach that considers how cultural frameworks shape the patient’s understanding of illness and death. For healthcare providers, this means attending to the content of disclosure as well as the manner in which it is delivered, as some cultures may prefer gradual conversation, and involvement or disinvolvement of family members and spiritual leaders. Open, early conversations are recommended to foster a better alignment between medical protocols and patient values [27,39].

In cultures where collectivism and relational identity are emphasized (i.e., Asian, Middle Eastern, Southern European, and Latin American communities), families may act as gatekeepers of information, deciding whether and how much of the prognosis should be shared [7,9,12,14-19,23,24,28,29]. This practice stands in contrast to individualistic frameworks common in Western healthcare systems, where truth disclosure is seen as a right of the patient and a necessary condition for informed consent. Certain studies [15,23,24] further challenged the Western assumption that delegation of decision-making to the family constitutes a loss of autonomy. Rather, in these cultural contexts, family involvement in disclosure is seen as an extension of the patient’s values, i.e., an expression of care, duty, and shared responsibility. As such, withholding or staging the disclosure of bad news is not necessarily considered deceptive or unethical, but rather a compassionate and respectful effort to shield loved ones and elders from distress.

Interestingly, some physicians themselves report feeling ill-equipped to navigate these conversations across cultural contexts. Providers may fear damaging the therapeutic alliance or misjudging a patient’s readiness to receive a terminal diagnosis [18,40]. Moreover, institutional policies that mandate full disclosure, such as those in the U.S., Canada, and some Northern European countries, often leave little room for flexible, culturally attuned communication. In contrast, healthcare settings in Asia, the Middle East, and some Southern European countries maintain more paternalistic models, with disclosure contingent on family input or physician discretion. A rigid application of Western disclosure norms may inadvertently produce harm, especially when patients come from backgrounds where indirect communication is a marker of empathy rather than evasion.

This divergence underscores a broader ethical tension: Is respect for persons best upheld through complete transparency, or through relational sensitivity to how information is received and processed? From a clinical standpoint, these findings suggest the need for early, individualized conversations about disclosure preferences, ideally before a serious illness prognosis. Rather than assuming that full disclosure is always desired or appropriate, providers should ask patients (and families, when culturally relevant) about their values and expectations. Incorporating cultural humility into communication training and providing clinicians with frameworks to negotiate cultural differences without stereotyping will be essential to improving the quality of end-of-life conversations.

Ultimately, truth disclosure should not be reduced to a binary option of telling or not telling. It is an ongoing, relational process that must balance ethical commitments, cultural context, and clinical judgment. Recognizing this complexity and responding with flexibility will be key to delivering respectful, patient-centered care in an increasingly multicultural world.

Patient autonomy: Patient autonomy entails a patient’s individual right of freedom in medical decision-making without undue influence or manipulation of others, such as healthcare providers, family, friends, and caregivers [40]. Across the studies included in this review, themes of patient autonomy and truth-disclosure frequently overlapped. Patient autonomy was often tied to truth disclosure, as access to accurate information was a prerequisite for autonomous decision-making. In turn, truth disclosure was frequently tied to family involvement and subsequent loss of patient autonomy, particularly in cultural contexts where families mediate communication between patients and providers.

In many Asian, Middle Eastern, and Southern European countries, families had central roles in end-of-life decision-making, often filtering information or making decisions on the patient’s behalf. While this may appear to undermine patient autonomy from a Western perspective, it is seen more as a sign of respect and protection for the patient and their family [9,16,18,19]. In contrast, Western healthcare systems, such as those in the U.S. and Canada, have legislative rules that safeguard patients’ right to actively participate in their healthcare and make informed decisions, based on broader historical values of self-determination [18,40]. However, there is growing evidence that some healthcare providers from traditionally collectivist societies (i.e., parts of Asia and Southern Europe) are increasingly adopting end-of-life care approaches that reflect Western values of patient autonomy [9,16].

The constantly evolving perspectives on patient autonomy in end-of-life care have important implications for clinical practice and policy development. It is crucial for healthcare providers to respect patients’ preferences for treatment and decisions about end-of-life care, whether it be individually or in consultation with family. To do so, healthcare providers can employ value-based conversations about patient autonomy preferences prior to discussing any medical information. Meanwhile, for policymakers, the findings from these studies point to the need for more flexible end-of-life care models that can accommodate a range of patient autonomy preferences. Ultimately, a broader comprehension of what patient autonomy means in different cultures must be understood by both healthcare providers and policymakers in order to ensure best care practices in end-of-life care across diverse populations.

Perspectives of death and dying: End-of-life decision-making is a dynamic and collaborative process involving patients, families, caregivers, and health-care providers and requires sensitivity to patients’ values, goals, and lived experiences [41]. Across studies in this review, culture emerged as a key determinant shaping not only how decisions are made, but how death itself is understood and approached [19,32]. Cultural frameworks influence patients’ perceived meaning of suffering, and the acceptability of interventions like palliative care or therapy limitation. For example, in Western and Northern European contexts, death is often framed through a medicalized lens that emphasizes symptom management, prognostic clarity, and patient control [5,20]. Within this framework, dying is approached as a process to be anticipated, discussed openly, and managed with clinical precision. Patients are encouraged to participate in autonomous decision-making that prioritizes transparency, advance directives, and palliative comfort. In contrast, many Asian, Middle Eastern, Latin, and Southern European cultures approach death as a spiritual passage, communal experience, or taboo subject [14,16,21,23,32]. Some patients and families may view accepting palliative care or halting curative treatment as “giving up,” conflicting with values of endurance, divine will, or familial duty [19,21,32]. Such stark differences in preference for end-of-life care have critical implications for clinicians. Standardized approaches to end-of-life care, care, particularly those emphasizing early advanced care planning, patient-led decision-making, and the transition to comfort-focused treatment, may be misaligned with patients’ cultural expectations. Clinicians must therefore move beyond protocol-driven communication to engage in value-based conversations that elicit patients’ and families’ beliefs about illness, dying, and medical authority. This requires cultural humility and institutional support, including access to interpreters, spiritual care, and culturally informed providers.

From a policy standpoint, these findings call for flexible models of end-of-life care that can accommodate a range of beliefs and preferences. For example, policies might enable shared decision-making with family proxies when requested by the patient, or allow for gradual disclosure of prognosis when aligned with cultural values. Likewise, training healthcare providers to recognize cultural expressions of distress, understand diverse spiritual frameworks, and navigate moral discomfort around care decisions can improve the responsiveness of health systems to multicultural populations.

Ultimately, integrating cultural understandings of death and dying into end-of-life care planning is not simply a matter of sensitivity, but it is a matter of quality and equity. Health systems must not only provide choices but ensure those choices are intelligible, meaningful, and respectful within the patient’s worldview.

Perspectives From Diasporas in the Western World

The presence of diasporas in Western countries has introduced complexities in healthcare delivery. As immigrant populations navigate unfamiliar healthcare systems, their experiences at the end of life are shaped not only by cultural beliefs but also structural vulnerabilities, including immigration status, language barriers, and systemic discrimination [23,24,29,32]. These unique challenges can influence access to care and trust in Western healthcare institutions for quality end-of-life support.

For instance, for undocumented patients, fear of deportation for themselves or their families may deter them from seeking hospital care altogether [24]. Reports of deportation following healthcare visits have heightened anxiety and contributed to mistrust in the healthcare system [24,42]. These concerns can discourage patients from disclosing critical information or accepting necessary interventions. While healthcare providers may be limited in their ability to address systemic immigration policies, they can still present patients with culturally informed end-of-life care practices that may foster trust with the patient and allow for open communication between providers and patients.

Furthermore, language barriers present additional obstacles. While providing care in the patient’s preferred language can improve satisfaction and trust, it is not always sufficient to ensure cultural competence. Physicians may still hold implicit biases or lack knowledge of appropriate cultural frameworks [43]. As Smith et al. argue, linguistic fluency does not replace the need for a deeper understanding of a patient’s cultural values that inform end-of-life care preferences. Thus, effective care in multicultural contexts may require coordination with bicultural liaisons and translators who can bridge linguistic and cultural divides. This highlights the distinction between cultural competence as a technical skill and cultural humility as an ongoing reflective practice [43,44].

From a policy perspective, these findings point to the need for system-level interventions that reduce disparities in access and care quality for immigrant populations. This includes investing in interpreter services, legal and social support networks within hospitals, and community partnerships that build trust among marginalized groups [24,32,43,44]. Clinicians, in turn, must be equipped to recognize how systemic inequities intersect with culture to shape patient behavior, expectations, and vulnerabilities at the end of life. Without such awareness, there is a risk that cultural misunderstandings may be misinterpreted as noncompliance or apathy, rather than as rational responses to structural risk and exclusion [43].

Ultimately, ensuring equitable end-of-life care for diasporic and immigrant communities requires more than cultural sensitivity. It calls for clinicians and health systems to respond to the broader social and political contexts that shape how patients experience illness, end-of-life care, and death.

Practical Implications for Healthcare and Policy Development

The findings of this review point to a gap between cultural diversity in patient populations and the standardization of end-of-life care policies in many Western systems. While earlier sections detailed how cultural beliefs shape preferences for disclosure and decision-making, the broader implication is that a one-size-fits-all approach to care delivery is insufficient.

Healthcare institutions must go beyond generic cultural competence training and invest in structural supports, such as interpreter services, bicultural liaisons, and community partnerships, that enable flexible, culturally attuned care. For clinicians, this may include further training on culturally informed care, especially in multicultural areas. This will foster value-based dialogue that respects cultural variation without relying on assumptions or stereotypes.

From a policy perspective, integrating cultural humility into institutional protocols and revisiting rigid autonomy-based mandates can help bridge the gap between policy ideals and patient realities. Future efforts should also consider how to train and support providers working at the intersection of clinical care, multicultural contexts, and systemic inequity.

Recent Advancements in End-of-Life Care

Over the past few years, there have been several advancements in the approach and practice of end-of-life care. Such advancements include the early integration of palliative care in oncology and critical care settings, tele-palliative care models, and ACP innovations.

Early integration of palliative care in oncology and critical care settings: Approaching oncology and critical care with an early integration of palliative care alongside active treatment has been shown to improve quality of life. Tailoring palliative care to consider global differences in cancer epidemiology, symptomatology, and cultural backgrounds can yield improved symptom control, decreased futile treatments, and increased patient satisfaction. However, such early integration may not be widely available globally due to politics, economic reasons, or stigma, presenting a challenge to patients in such regions of the world with advanced cancer [45,46].

Tele-palliative care models: Palliative care delivered via telehealth has been effective in improving the quality of life in patients, especially for patients residing in rural communities and/or patients with limited mobility and lack of transportation for medical services. This format of delivering end-of-life care has accelerated post-COVID-19 pandemic. The health-care platform ensures continuity of care by allowing the ability to manage symptoms efficiently in a timely manner and conduct family meetings in times when family members cannot be physically present [47]. However, tele-palliative care continues to present challenges in terms of digital literacy and access to technology among certain demographics globally in resource-limited areas [45,47].

ACP innovations: ACP entails a discussion and documentation of the patient's future healthcare wishes, values, and life goals [48]. ACP is a vital component of palliative care, as it honors patient autonomy. The integration of culturally sensitive tools into ACP discussions with patients has ensured that patients' cultural perspectives on their end-of-life care are acknowledged and respected in practice [47,48].

Limitations

This review presents several limitations that may affect the interpretation and generalizability of its findings. First, many of the included studies relied heavily on small sample sizes and qualitative methods [6,8,12,15,25,29]. While qualitative approaches are essential for exploring cultural nuance and subjective experience, their findings are context-dependent and may not reflect broader population trends. The limited use of large-scale, quantitative data restricts the capacity to assess the prevalence or magnitude of cultural influences on end-of-life care. Future research would benefit from mixed-methods designs or nationally representative surveys to further investigate these insights and enhance validity.

Second, the review was limited to studies published in English due to resource and personnel constraints. This introduces a language bias, as this may have excluded literature from non-English-speaking regions. Given the global focus of this review, the exclusion of non-English publications likely narrowed the cultural scope of perspectives analyzed, particularly from non-Western and underrepresented communities. This may have led to an incomplete understanding of global diversity in end-of-life values and practices. Moreover, a formal gray literature search was not conducted (i.e., OpenGrey, Institute of Scientific and Technical Information-National Centre for Scientific Research, Nancy, France, or government databases), which further limits the scope of perspectives analyzed.

Third, while the review includes attention to some ethnic minority populations, such as the Romanian Roma communities, and Italian and Latino immigrants, the experiences of other migrant communities (i.e., African, South American, and Oceanic populations) remain underexplored. This reflects a broader gap in the literature regarding culturally specific end-of-life preferences for certain populations. Future research should aim to include these perspectives to avoid the exclusion of marginalized voices in palliative and end-of-life care literature.

Fourth, the analysis could be further enriched by deeper contextualization of how national health-care systems influence end-of-life decision-making. Factors such as the availability and accessibility of palliative care, the structure of national insurance systems, and country-specific legal frameworks, such as euthanasia laws in Belgium and the Netherlands, play a significant role in shaping patients' treatment preferences and provider behavior. The interaction between structural systems and cultural values remains an important area for future investigation. Moreover, this review did not account for region-specific sociopolitical or environmental factors that may influence how death and dying are understood. Cultural practices are often shaped not only by heritage but also by contemporary policy environments, legal norms, and historical legacies. The omission of such contextual analysis, especially regarding differences across and within countries, may have limited the explanatory depth of the review.

Thus, while this review synthesizes key themes across diverse cultural contexts, limitations related to language scope, methodological imbalance, minority representation, and policy contextualization point to several directions for future research. Addressing these gaps will be critical to developing a more holistic and equitable understanding of end-of-life decision-making across global populations.

Future research

While current literature provides valuable insight into how cultural differences shape patients’ experiences and decisions in end-of-life care, further research is needed to explore these dynamics across diverse global contexts. As societies become increasingly multicultural, it is essential to examine how cross-cultural communication influences palliative and hospice care delivery. Future qualitative studies should include both patient and healthcare provider perspectives, using methods such as semistructured interviews, focus groups, and ethnographic approaches to capture the complexity of cultural values and decision-making processes. Research that examines how clinicians interpret and navigate cultural norms in real-world settings, particularly in relation to institutional policies or systemic barriers, would be especially valuable. Moreover, investigating how intersecting factors such as immigration status, language proficiency, and health-care access affect end-of-life care across regions can inform more equitable models of care, especially in Western contexts. Ultimately, there is a need for interdisciplinary, cross-national research that integrates qualitative and quantitative methods to support the development of culturally responsive, patient-centered end-of-life practices worldwide.

## Conclusions

End-of-life care, palliative care, and hospice care are highly impacted by cultural nuances. The role of culture within the intersection of the patient, the patient’s family, and the physician in end-of-life decision-making is unique from other aspects of healthcare. Future efforts should focus on embedding cultural competence into healthcare policies and clinical guidelines to ensure culturally sensitive truth disclosure and decision-making in end-of-life care. Medical education and ongoing training must prioritize cultural awareness and effective communication to equip providers for diverse patient populations. Additionally, fostering interdisciplinary collaboration and developing patient- and family-centered care models that respect cultural values can enhance trust, autonomy, and meaningful decision-making. Further research is needed to identify specific cultural influences on end-of-life preferences and to evaluate interventions that improve culturally tailored care. Utilizing technology, such as translation services and culturally adapted decision aids, may also help bridge communication gaps. Thus, incorporating cultural factors as a central element in end-of-life care is essential for ensuring compassionate, individualized treatment and advancing equity in healthcare delivery on a global scale.
